# A nomogram to predict postoperative deep vein thrombosis in patients with femoral fracture: a retrospective study

**DOI:** 10.1186/s13018-023-03931-1

**Published:** 2023-06-27

**Authors:** Linqin Wu, Bo Cheng

**Affiliations:** Department of Anesthesiology, The First Affiliated Hospital of Chongqing Medical University, Chongqing, China

**Keywords:** Deep venous thrombosis, Risk factor, Femoral fracture, Nomogram model, The Caprini score

## Abstract

**Objective:**

The implementation of more active anticoagulant prevention and treatment measures has indeed led to a significant reduction in the incidence of perioperative deep vein thrombosis (DVT) among patients with bone trauma. However, it is important to note that despite these efforts, the incidence of DVT still remains relatively high. According to the Caprini score, all patients undergoing major orthopedic surgery were defined as the high-risk group for DVT. Stratifying the risk further within high-risk groups for DVT continues to present challenges. As a result, the commonly used Caprini score during the perioperative period is not applicable to orthopedic patients. We attempt to establish a specialized model to predict postoperative DVT risk in patients with femoral fracture.

**Methods:**

We collected the clinical data of 513 patients undergoing femoral fracture surgery in our hospital from May 2018 to December 2019. According to the independent risk factors of DVT obtained by univariate and multivariate logistic regression analysis, the corresponding nomogram model was established and verified internally. The discriminative capacity of nomogram was evaluated by receiver operating characteristic (ROC) curve and area under the curve (AUC). The calibration curve used to verify model consistency was the fitted line between predicted and actual incidences. The clinical validity of the nomogram model was assessed using decision curve analysis (DCA) which could quantify the net benefit of different risk threshold probabilities. Bootstrap method was applied to the internal validation of the nomogram model. Furthermore, a comparison was made between the Caprini score and the developed nomogram model.

**Results:**

The Caprini scores of subjects ranged from 5 to 17 points. The incidence of DVT was not positively correlated with the Caprini score. The predictors of the nomogram model included 10 risk factors such as age, hypoalbuminemia, multiple trauma, perioperative red blood cell infusion, etc. Compared with the Caprini scale (AUC = 0.571, 95% CI 0.479–0.623), the calibration accuracy and identification ability of nomogram were higher (AUC = 0.865,95% CI 0.780–0.935). The decision curve analysis (DCA) indicated the clinical effectiveness of nomogram was higher than the Caprini score.

**Conclusions:**

The nomogram was established to effectively predict postoperative DVT in patients with femoral fracture. To further reduce the incidence, more specialized risk assessment models for DVT should take into account the unique risk factors and characteristics associated with specific patient populations.

## Background

The bone trauma patients are considered to be at a high risk for developing deep vein thrombosis (DVT). The incidence of DVT was as high as 69% in patients with lower limb bone trauma who had not previously received thromboprophylaxis [1]. In recent years, with the implementation of more active prevention and treatment measures based on anticoagulation, the incidence has significantly decreased to approximately 10% [2, 3]. However, this has not changed the status quo that the incidence of DVT in patients with bone trauma is still relatively high. Until the more optimized individualized prevention strategies are explored, early identification of high-risk patients plays a crucial role in reducing the incidence of DVT. Therefore, the risk assessment of DVT in bone trauma patients should be the focus of current research.

Currently, the Caprini score is the most commonly used model for predicting postoperative venous thromboembolism (VTE) in surgical patients. This model has been validated in in various surgical patient populations including critically ill surgical patients [4], plastic and reconstructive surgery patients [5], otolaryngology [6], gynecologic oncology [7], general surgery, vascular surgery, and urology [8]. In terms of bone trauma patients, the Caprini score is recognized and recommended by most relevant guidelines for evaluating the risk of VTE. These guidelines include the American College of Chest Physicians (ACCP) guidelines [9], the Chinese expert consensus on perioperative VTE prevention in orthopedic trauma patients [10], the Chinese guidelines for VTE prevention in major orthopedic surgery [11], etc. However, we should reevaluate the applicability of the Caprini score for DVT risk assessment in patients with bone trauma. Firstly, all patients undergoing major orthopedic surgery were classified as DVT high-risk group if their score is equal to or greater than 5. We are unable to use the Caprini score for a more granular risk stratification of these patients. Secondly, the Caprini score has not been validated in orthopedic patients [12], and its effectiveness has not been confirmed by more studies. The Capini score in Asian patients remains to be further validated [13]. Shuster et al. [14] reported that the Caprini score was not suitable for trauma patients. The cut-off point of Caprini value is not consistent in trauma patients compared with other patients [15]. Additionally, Bateman et al. [16] demonstrated that it was ineffective for risk stratification in patients undergoing total joint replacement.

In conclusion, the Caprini score, which is commonly used in the perioperative period, may not be suitable for accurately assessing the risk of DVT in patients with bone trauma. Indeed, achieving a more accurate assessment of the risk of DVT in high-risk patients with bone trauma continues to be a challenge.. In order to identify high-risk patients as early as possible and further reduce the incidence of DVT, it is particularly important to establish a specialized model for patients with bone trauma. In recent years, most studies on DVT risk assessment focus on risk factors rather than risk prediction models. Therefore, we integrated the associated risk factors to establish a comprehensive model for predicting the risk of postoperative DVT in patients with femoral fracture. The development of this nomogram could probably provide a valuable tool for doctors to identify high-risk DVT patients and formulate appropriate prevention strategies promptly.

## Materials and methods

### Study subjects

A retrospective study was performed on patients undergoing femoral fracture surgery in our hospital from May 2018 to December 2019. This study has been approved by the Ethics Committee of the First Affiliated Hospital of Chongqing Medical University (2019–277) and the Chinese Clinical Trial Register (ChiCTR2000035103).

Inclusion criteria: (a) traumatic fracture; (b) aged over 18 years old; (c) complete clinical data; and (e) Doppler ultrasonography performed on both lower limbs. Exclusion criteria: (a) concomitant blood system disease or coagulation disorder; (b) long-term use of anticoagulants because of the history of thrombosis; (c) pregnancy; and (d) history of old thrombosis.

According to the results of lower extremity Doppler ultrasonography, the included subjects were divided into postoperative DVT group and non-DVT group. The patients in the DVT group included in this study were as follows: (a) patients with DVT after surgery and (b) patients with preoperative DVT that progressed after surgery.

### Collection of clinical data

The occurrence of lower extremity DVT was the grouping criterion for this study. It is widely acknowledged that ultrasound examination of veins is considered the gold standard for diagnosing DVT [17]. The pulsed Doppler ultrasonography was conducted on the veins of both lower extremities of the patients before and after surgery using C5-1 linear probe and IU22 system (Philips ATL, Bothwell, WA, USA).

In reference to relevant literature, risk factors that may be associated with DVT were considered as potential model predictors. The variables that may be associated with DVT were as follows: (a) gender, age, body mass index (BMI), smoking history, drinking history, and other basic information; (b) comorbidities including diabetes, hypertension, coronary heart disease, hyperlipidemia, hepatic disease, nephropathy, pulmonary diseases, chronic obstructive pulmonary disease (COPD), malignant tumor, hypoproteinemia, anemia, etc. (c) American Society of Anesthesiologists (ASA) classification; (d) trauma and surgery factors including high energy trauma, open fracture, compound fractures, comminuted fracture, shock, time from trauma to admission, use of tourniquets, anesthesia, intraoperative blood loss, perioperative red blood cell (RBC) infusion, intraoperative blood loss, etc. (e) anticoagulation therapy including preoperative anticoagulation, time from admission to anticoagulation, trauma to anticoagulation, postoperative anticoagulation, etc. (f) laboratory test indexes including c-creative protein (CRP) level, serum creatinine (Crea) level, serum urea level, albumin (Alb), white blood cells (WBC) count, neutrophil percentage (NEUT%), hemoglobin (Hb) level, platelets (PLT), lymphocyte percentage (LYM%), prothrombin time (PT) level, prothrombin time ratio (PTR) level, international normalized ratio (INR) level, prothrombin time activity (PTA) level, activated partial thromboplastin time (APTT) level, thrombin time (TT) level, fibrinogen (Fbg) level, D-dimer (DD) level, fibrinogen degradation product (FDP) level and lactic acid (lac) level.

### Statistical analysis

Univariate logistic regression analysis was performed on the above variables of the Study subjects. Statistically significant variables were included in multivariate logistic regression analysis. Finally, independent risk factors were obtained by logistic regression analysis using IBM SPSS Statistics 26.0 software (IBM Corporation, Armonk, NY, USA). The statistical test level was set to *P* < 0.05. Further, the forest plots were drawn with GraphPad Prism (version 8.00, GraphPad Software, San Diego, CA, USA) to visualize the results of the regression analysis.

Ultimately, the establishment of the nomogram model was based on independent risk factors derived from logistic regression analysis. R software was used to establish the nomogram model of risk assessment using the RMS package. Bootstrap method was applied to the internal validation of the nomogram model. The discriminative capacity of nomogram was evaluated by receiver operating characteristic (ROC) curve and area under the curve (AUC). The calibration curve used to verify model consistency was the fitted line between predicted and actual incidences. The clinical validity of the nomogram model was assessed using decision curve analysis (DCA) which could quantify the net benefit of different risk threshold probabilities [18]. Besides, we analyzed the Caprini scores of the included subjects and compared the performance of the nomogram model with the Caprini score in terms of DVT risk assessment.

## Results

A total of 513 patients with femoral fracture were included in this study. The included study subjects were 194 males and 319 females with a mean age of 71.5 ± 18.4 years (19–112 years). According to the grouping criteria, there were 91 patients (17.7%) in the DVT group and 422 patients (82.3%) in the non-DVT group.

### The Caprini score and incidence of DVT

**Fig. 1:**
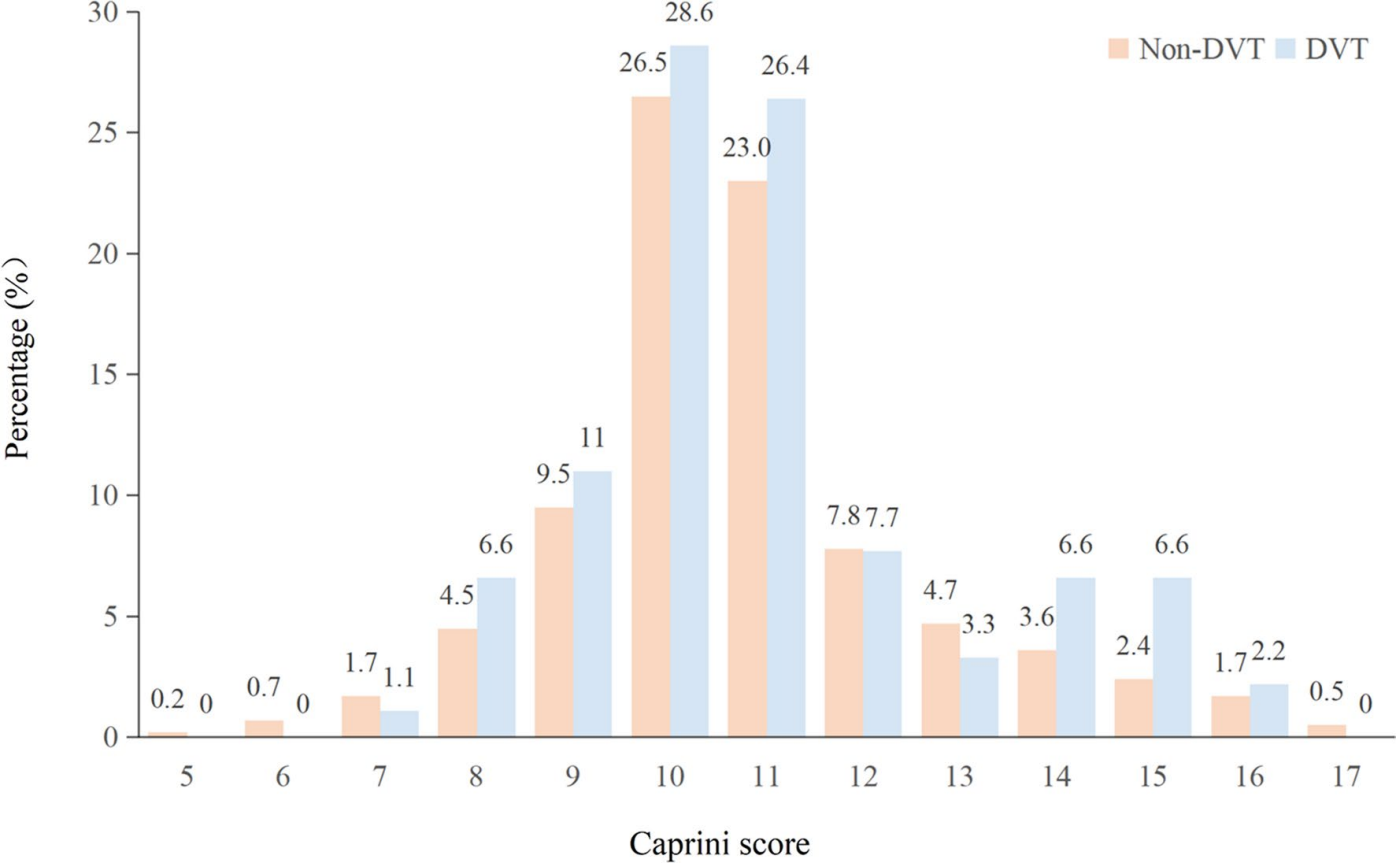
The Caprini scores of femoral fracture patients

The Caprini scores of subjects ranged from 5 to 17 points (Fig. 1). Obviously, the majority of patients scored in the middle range of 10 to 11. The scores of patients in the DVT group ranged from 6 to 16 points, with 10 points and 11 points accounting for 28.6% (26/91) and 26.4% (24/91), respectively. The non-DVT group scored 5 to 17 points, of which 26.5% (111/422) were 10 and 23.0% (97/422) were 11. There were more patients in the DVT group than in the non-DVT group in the 8–11 and 14–16 score intervals. In the rest of the score ranges, the non-DVT group accounted for a larger proportion than the DVT group.

**Fig. 2:**
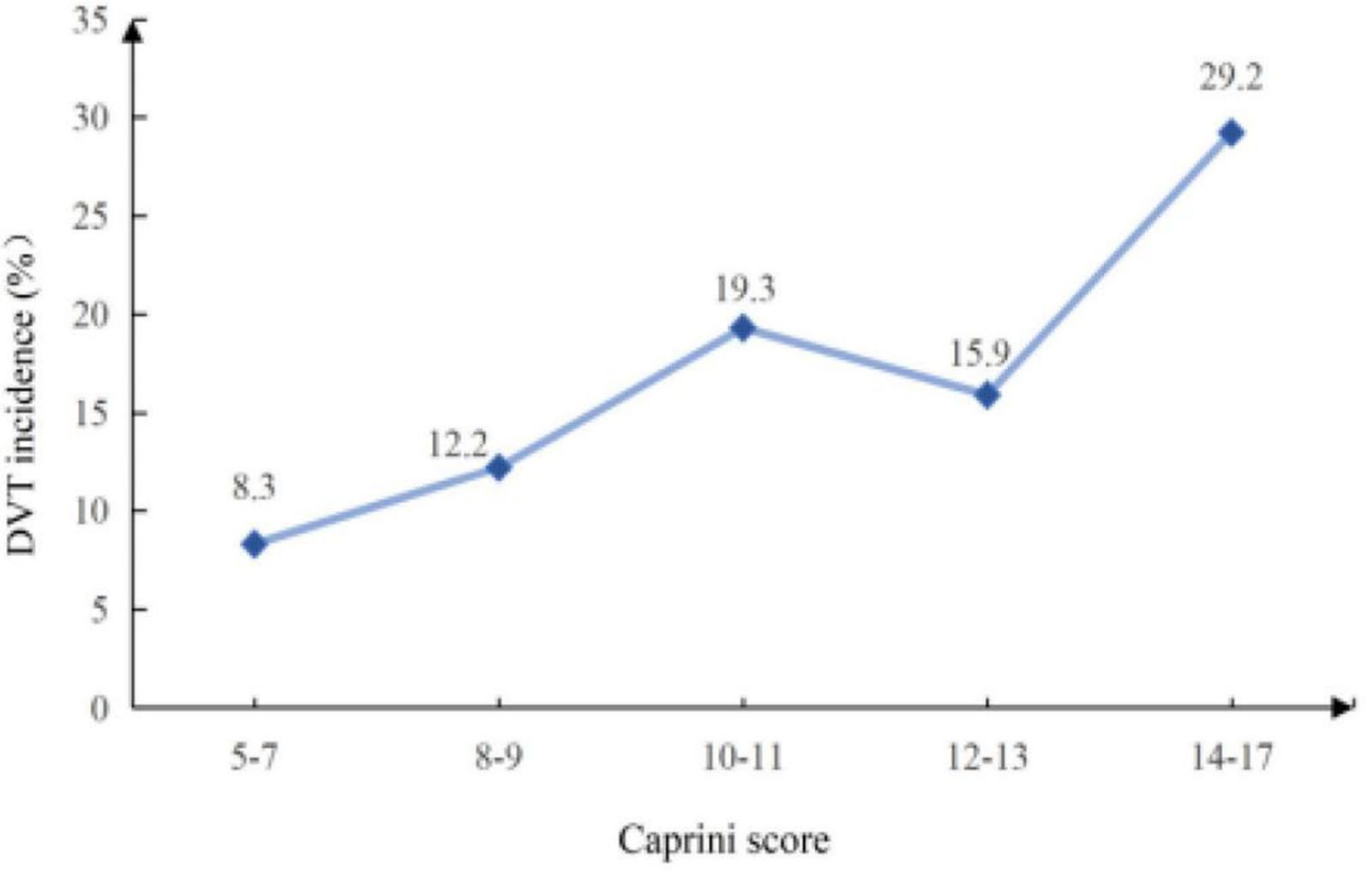
The DVT incidence in different Caprini score groups

The incidence of postoperative DVT in patients with femoral fracture in this study was 17.7% (91/513). Figure 2 presents the incidence of DVT in different Caprini score intervals. The highest incidence of DVT was at 14–17 points, and the lowest was at 5–7 points. The incidence rates of the two score intervals were 29.2% (14/48) and 8.3% (1/12), respectively. It should be noticed that the incidence of the 12–13 score interval was lower than that of the 10–11 score interval, so there was no positive correlation between the incidence of DVT and Capini score.

### Independent risk factors for postoperative DVT

The independent risk factors were obtained by logistic regression analysis of the relevant variables of the study subjects. The basic information, ASA classification, trauma and surgery factors, anticoagulation therapy and laboratory test indexes were included as relevant variables in this study.

#### Univariate logistic regression analysis

**Fig. 3:**
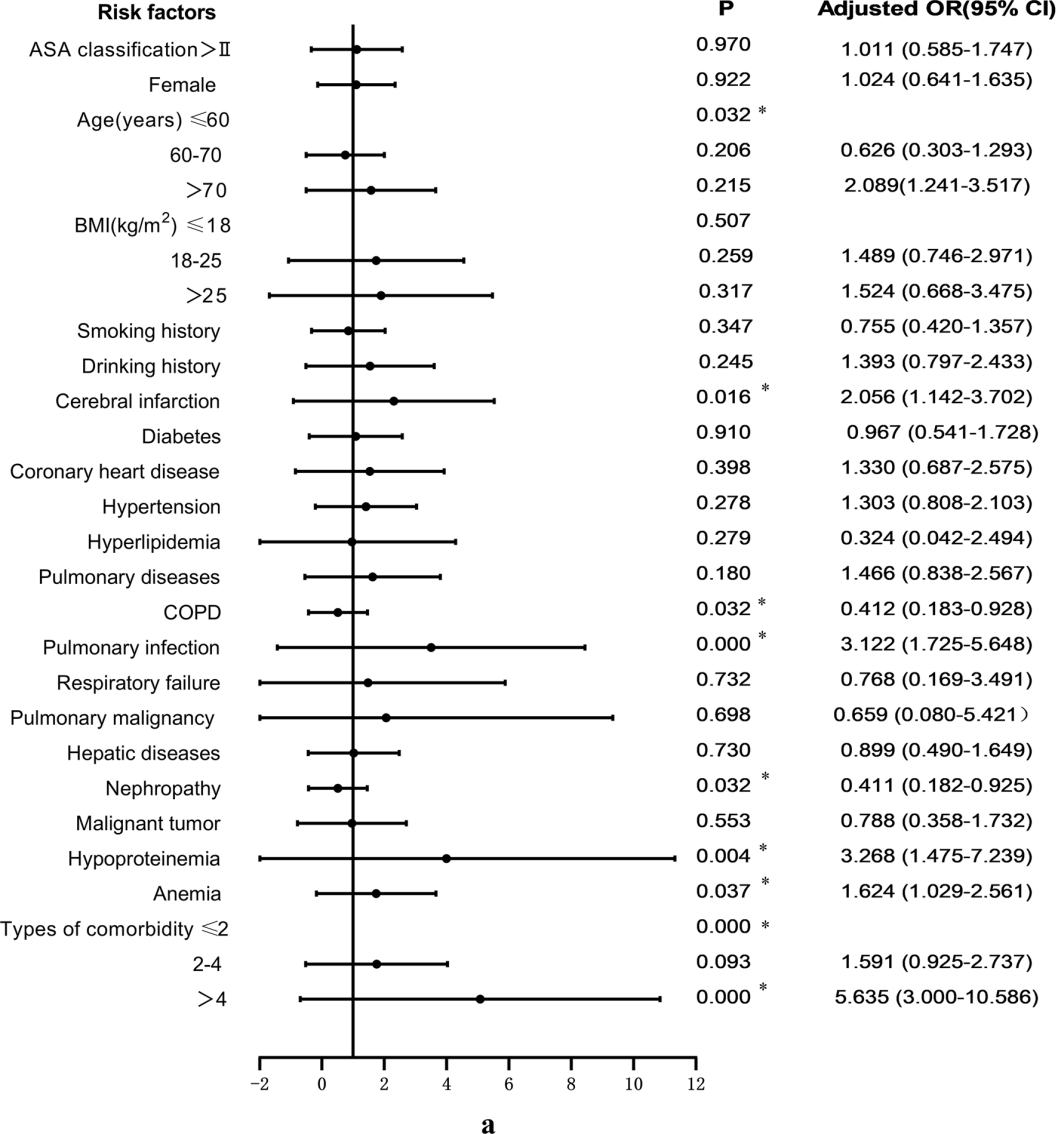

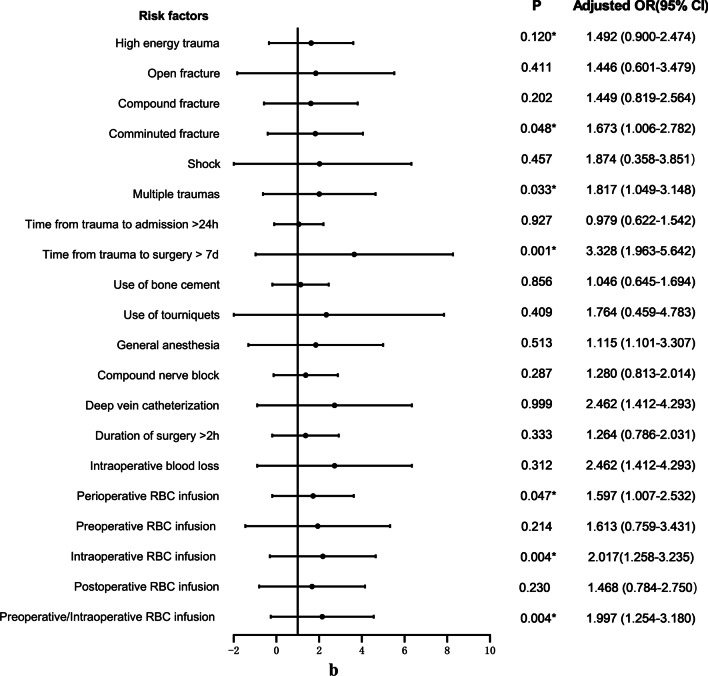

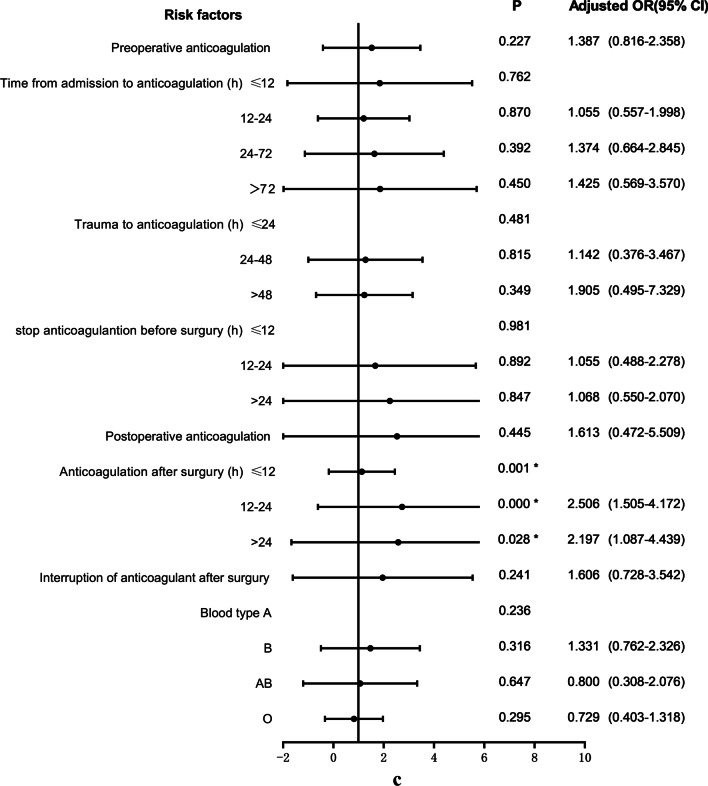

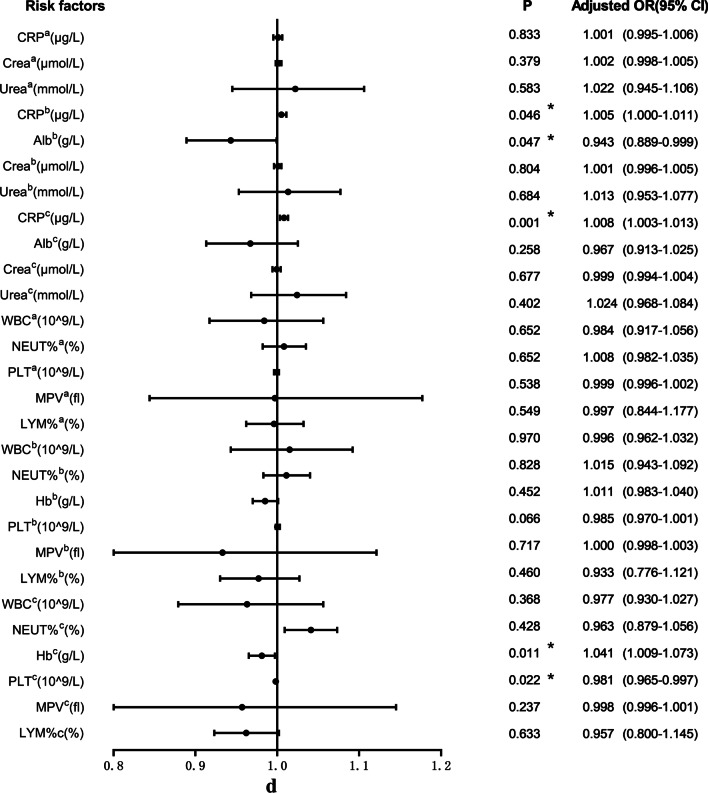

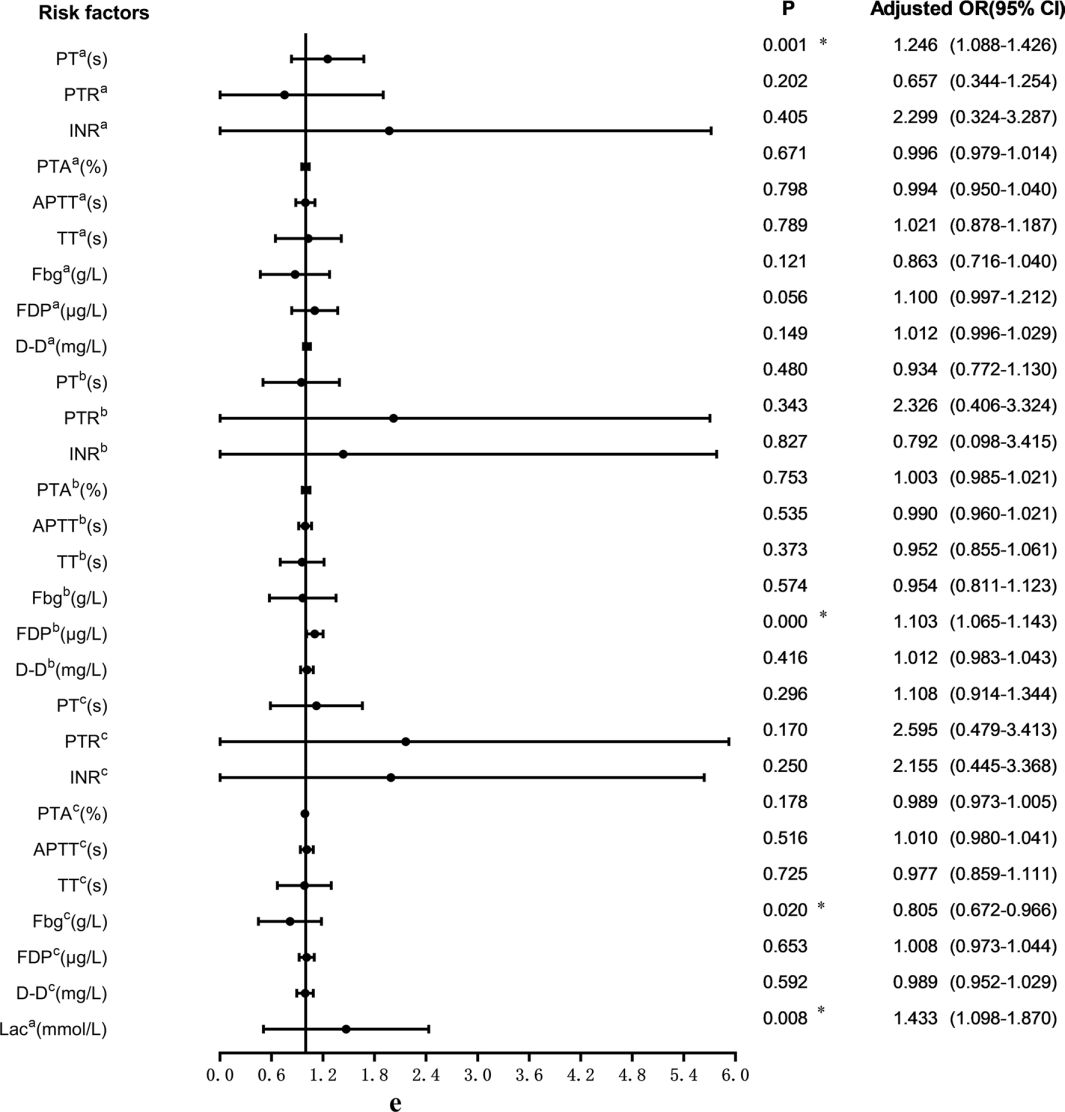
**a** Univariate logistic analysis of risk factors for postoperative DVT in patients with femoral fracture. **b** Univariate logistic analysis of risk factors for postoperative DVT in patients with femoral fracture. **c** Univariate logistic analysis of risk factors for postoperative DVT in patients with femoral fracture. **d** Univariate logistic analysis of risk factors for postoperative DVT in patients with femoral fracture. **e** Univariate logistic analysis of risk factors for postoperative DVT in patients with femoral fracture. Abbreviations: ^a^The index upon admission; ^b^The index 1 day after surgery; ^c^The index 3 days after surgery

17.7% (91/513) of patients with femoral fracture developed postoperative DVT. The relevant clinical data of patients in the non-DVT group and DVT group were included in the univariate logistic regression analysis, as shown in Fig. 3a–e. The variables that were statistically significant between the two groups included COPD (*P* = 0.032), pulmonary infection (*P* = 0.000), nephropathy (*P* = 0.032), hypoproteinemia (*P* = 0.004), anemia (*P* = 0.037), intraoperative RBC infusion (*P* = 0.004) and so on. At the same time, BMI (*P* = 0.507), diabetes (*P* = 0.910), coronary heart disease (*P* = 0.398), use of bone cement (*P* = 0.856) and other variables with *P* > 0.05 were not statistically significant.

#### Multivariate logistic regression analysis

**Fig. 4:**
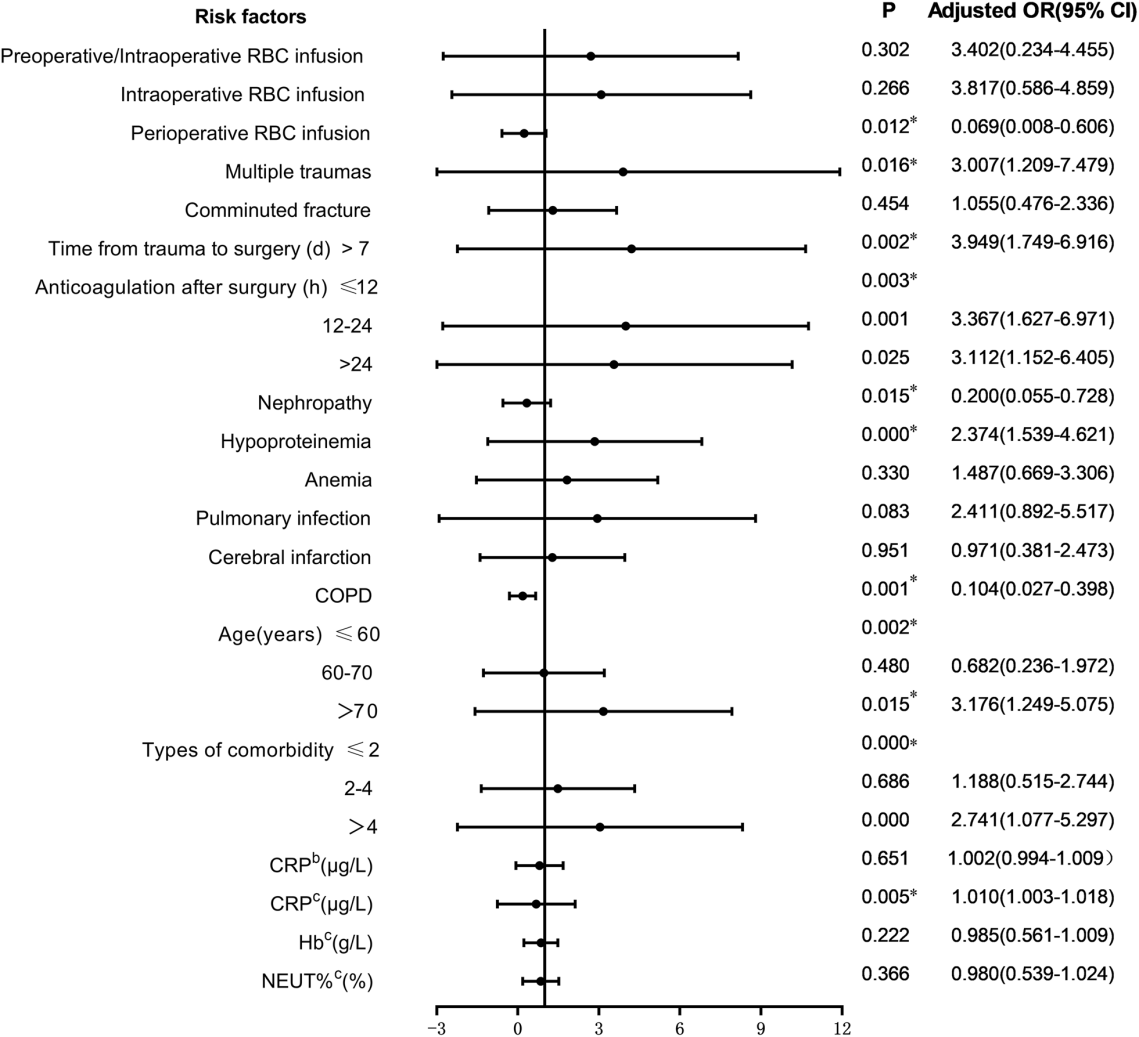
Multivariate logistic analysis of risk factors for postoperative DVT in patients with femoral fracture

The variables with *P* < 0.05 in univariate logistic regression analysis were conducted for multivariate logistic analysis (Fig. 4). The independent risk factors of postoperative DVT in patients with femoral fracture included perioperative RBC infusion (*P* = 0.012), multiple traumas (*P* = 0.016), time from trauma to surgery > 7d (*P* = 0.002), time to start anticoagulation after surgery (*P* = 0.003), nephropathy (*P* = 0.015), hypoproteinemia (*P* = 0.000), COPD (*P* = 0.001), age (*P* = 0.002), the number of comorbidity (*P* = 0.000) and CRP level on day 3 after surgery (*P* = 0.005).

### Establishment of the nomogram model

**Fig. 5:**
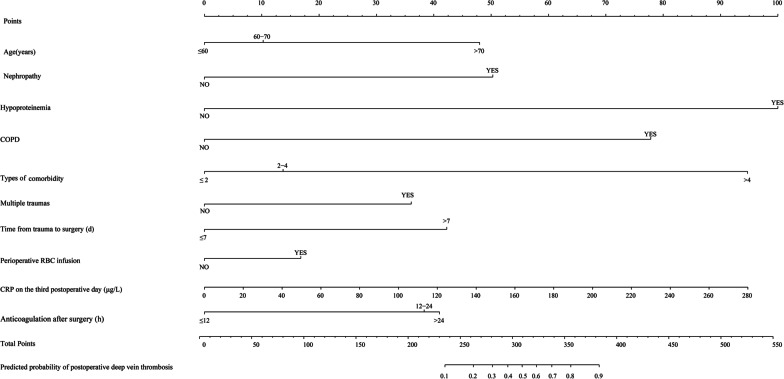
Predictive nomogram for postoperative DVT in patients with femoral fracture

According to the multivariate logistic analysis results of the study subjects, 10 independent risk factors were included in the establishment of the nomogram model (Fig. 5). The structure of the nomogram is shown in Fig. 5. The corresponding score for each predictor variable was obtained, and then the sum of these scores was the final total score. Eventually, the predicted probability corresponding to the total score was the risk of DVT in patients with femoral fracture after surgery. For example, a 65-year-old femoral fracture patient with hypoproteinemia, liver disease, and hypertension ( more than 2 kinds of comorbidities) received surgical treatment 8 days after trauma. The patient received perioperative RBC infusion, and then anticoagulation was started 12 h after surgery. In addition, CRP on postoperative day 3 was 180 ( μg/L). The sum of the scores for each predictor was 249 points, so the risk of postoperative DVT in this patient was 15%.

### Verification and comparison of the Nomogram model

#### Calibration

**Fig. 6:**
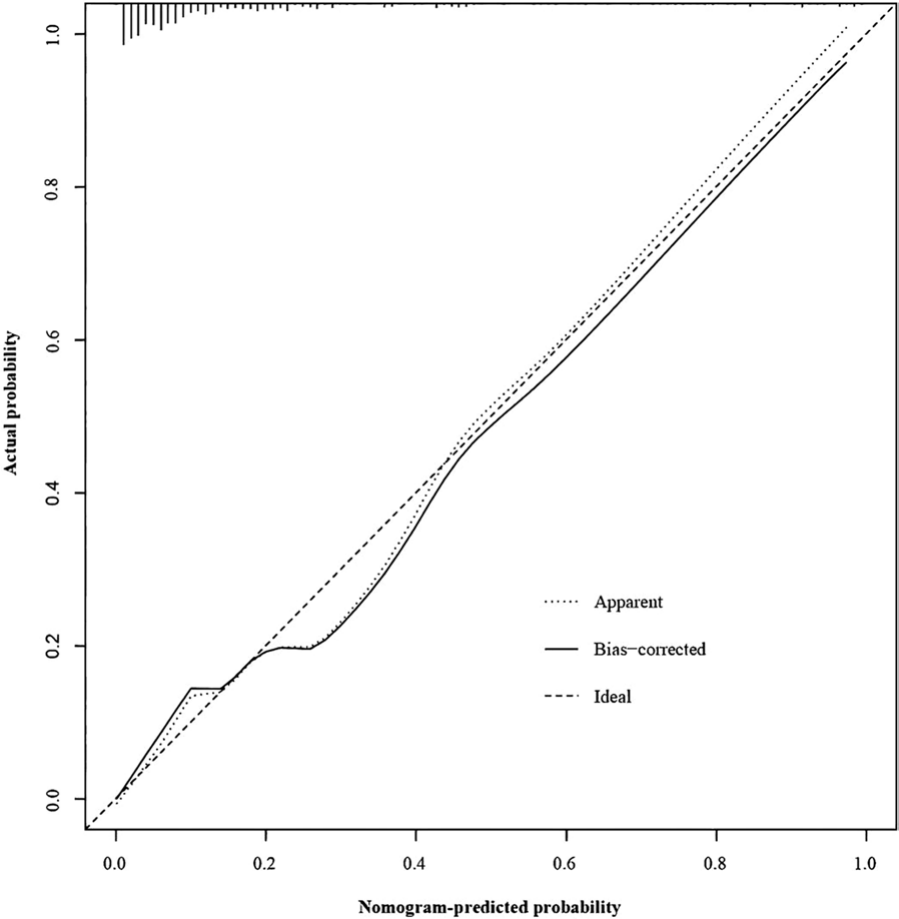
Calibration curve for nomogram of postoperative DVT in patients with femoral fracture

The nomogram model was internally validated as shown by the calibration curve for postoperative DVT nomogram prediction in patients with femoral fracture (Fig. 6). The X-axis represents the nomogram-predicted probability of DVT in patients with femoral fracture, and the Y-axis represents the actual probability of DVT. Obviously, the predicted probability of the nomogram was highly similar to the actual probability.

#### Distinguishing degree

**Fig. 7:**
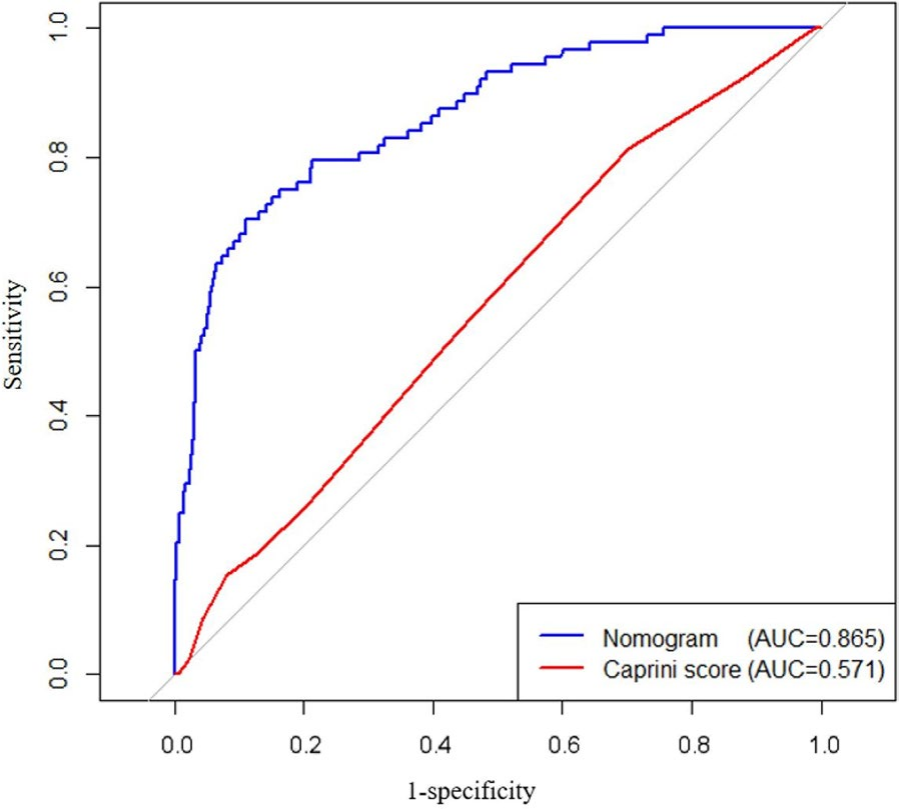
The ROC curve of the nomogram model and the Caprini score. Abbreviations: The AUC was used to evaluate the discrimination of the model. ROC, receiver operating characteristic; AUC, area under the curve

The nomogram predictive model had good discrimination because the ROC curve showed that the nomogram had an AUC value of 0.865 (95% CI 0.780–0.935) in Fig. 7. Further, all 513 patients in this study had Caprini scores ≥ 5 (Fig. 1), which should be defined as patients at high risk of DVT. The AUC of the Caprini score was 0.571 (95% CI 0.479–0.623), which was significantly lower than the 0.865 of the nomogram model (Fig. 7). In terms of patients with femoral fracture, it followed that the discrimination of the nomogram model was better than the Caprini score.

#### Clinical effectiveness

**Fig. 8:**
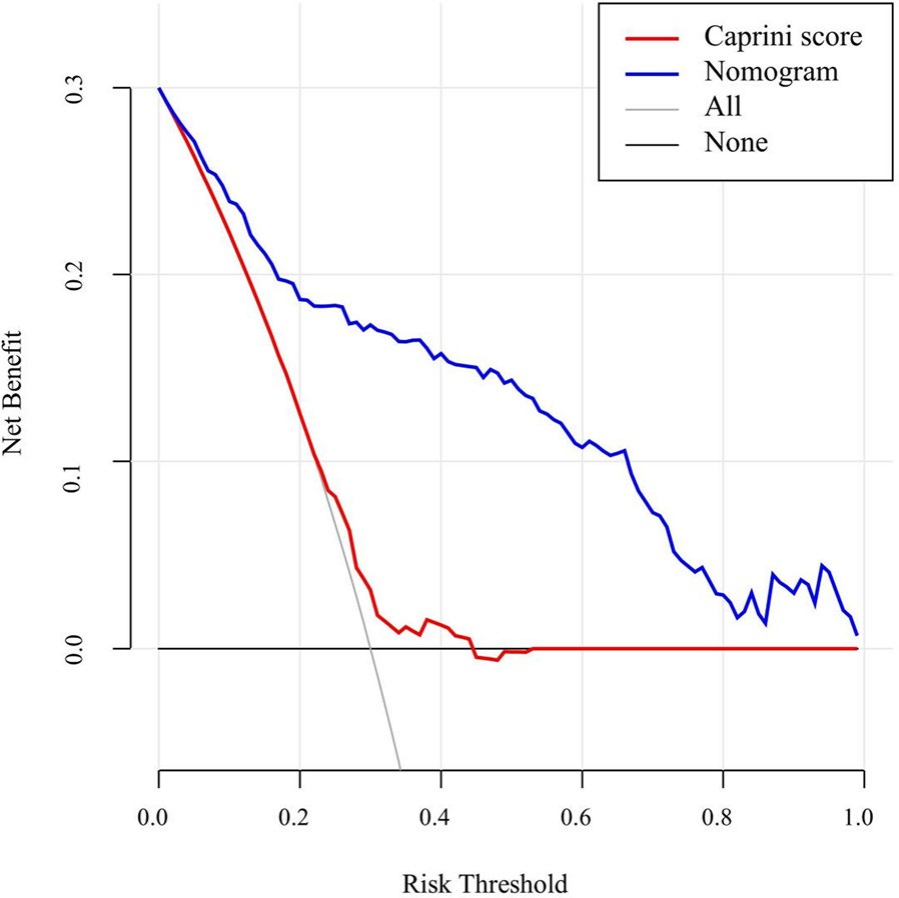
The decision curve analysis for nomogram and Caprini score to predict the risk of postoperative DVT in patients with femoral fracture

The decision curve analysis (DCA) was used to evaluate the availability and benefits of the prediction model, which illustrated in Fig. 8 showed that the net benefit of the nomogram model was higher in the threshold probability interval of 5–95%. The decision curve for the Caprini score was close to the "all" and "none" curves (Fig. 8), indicating that the Caprini score had little net benefit. As for risk of postoperative DVT for femoral fracture, the clinical effectiveness of nomogram model was higher than Caprini score.

## Discussion

The bone trauma patients are at high risk of DVT. In a prospective study of patients with lower extremity fractures receiving anticoagulation, the incidence of DVT in the affected lower extremity was 35% before surgery, which increased to 55% after surgery, and 40% at 1 month after surgery [19]. The incidence of postoperative DVT in patients with intertrochanteric fractures was 11.5% in a retrospective study of 1672 patients [20]. Ren et al. [1, 21] reported that the incidence of postoperative DVT in patients with femoral shaft fracture was 15.6%. The incidence of postoperative DVT in patients with femoral fracture in this study was 17.7% (91/513). Obviously, we are still facing a relatively high incidence of DVT in patients with bone trauma. In clinical practice, it is particularly important for doctors to identify patients at high risk of DVT as soon as possible.

The Caprini score has been widely used to assess DVT risk in perioperative patients [22]. In a review by Benjamin et al.[23], models for perioperative VTE risk assessment in surgical patients were compared and the 2005 version of the Caprini score was shown to be a valid model for assessing the risk of DVT in surgical patients. Based on the Caprini score, 513 patients with femoral fracture in the study had a Caprini score ranging from 5 to 17 and most of them scored 10–11 points. There were more patients in the non-DVT group than in the DVT group in the 5–7 points and 12–13 points interval. In this study, both the DVT group and the non-DVT group had Caprini scores ≥ 5, which were defined as high-risk patients [22]. It was not difficult to find that the Caprini risk assessment did not effectively distinguish between the two groups of patients. We further analyzed the incidence of DVT in different Caprini score intervals, with the highest incidence in 14–17 points and the lowest in 5–7 points. It is worth noting that the incidence of DVT in this study was not completely positively correlated with the level of Caprini score. In conclusion, the Caprini score is not suitable for assessing the risk of DVT in patients with bone trauma without high discriminative and predictive ability. Secondly, the Caprini risk assessment is an empirical scale model, which evaluates VTE risk through the total score of about 40 risk factors related to patients and surgery [12]. Although the original Caprini score has been revised several times including adding new risk factors or adjusting the weights of already included risk factors [22]. This experience scale model is usually based on a unary linear relationship between variables, and scores are weighted according to the relative risk of thrombosis reported in the documents. There might be several potential issues with the Caprini risk assessment. Firstly, other risk factors for DVT in patients with bone trauma were not included in the model, such as surgical methods, laboratory test indicators, etc. Secondly, the weight of related risk factors might be inconsistent among different disease groups. Finally, linear assessment tools could not accurately assess interactions between multiple correlated factors. Considering that the most commonly used Caprini risk prediction model is not suitable for patients with bone trauma and the limitations of the Caprini score, we need to further explore the specialized DVT risk assessment model for bone trauma patients. This is necessary for early identification of high-risk patients and individualized DVT prevention and treatment.

As far as the perioperative DVT risk assessment in patients with bone trauma is concerned, most of the related studies are based on the analysis of risk factors, and there is a lack of accurate prediction models for the individual risk of patients [24–28]. Univariate logistic regression analysis was performed on 106 related variables including basic information, comorbidities, trauma and surgery factors, anticoagulation therapy, and laboratory test indexes of the study subjects. Further, we included factors with statistically significant differences (*P* < 0.05) into multivariate logistic regression analysis. Finally, 10 variables including perioperative RBC infusion, time from trauma to surgery (d) and anticoagulation after surgery (h) were independent risk factors. These independent risk factors for DVT were incorporated into the establishment of the nomogram model. Unlike previous studies of risk factors, independent risk factors were obtained only by combining chi-square test, T test and other methods and univariate logistic regression analysis. The independent risk factors in this study were more accurately analyzed by univariate and multivariate logistic regression. We conducted internal validation of nomogram and the calibration curve showed good agreement between the predicted probability and the actual DVT probability. The AUC value of the ROC curve of the established nomogram model was 0.865, indicating that the model had good discrimination for the occurrence of postoperative DVT in patients with femoral fracture. The DCA of the model also showed that the nomogram model had good the availability and benefits. Based on the Caprini score and the corresponding incidence of DVT in patients with femoral fracture in this study, the Caprini score is not suitable for assessing the risk of DVT in these patients. At the same time, the ROC curve and decision curve showed that our established nomogram prediction model had more advantages than the Caprini score in predicting the risk of postoperative DVT in patients with femoral fracture. At present, nomogram models for VTE risk assessment include portal vein thrombosis after splenectomy for liver cirrhosis [29], venous thrombosis after metastatic spinal tumor [30], lower extremity DVT after acute stroke [31] and DVT during acute exacerbation of COPD [32], etc. For trauma patients, Ling et al. [15] used a machine learning model to establish a modified Caprini score model for evaluating trauma hospitalized patients. Peng et al. [33] reviewed the clinical data of 281 patients and constructed a risk assessment model for predicting lower extremity DVT in patients with multiple trauma, with an area under the ROC curve of 0.890. Lin et al. [34] established a DVT risk prediction model for patients with lower extremity fractures. We included only one type of bone trauma, which was more targeted than the above studies related to patients with bone trauma. Secondly, we considered possible potential predictors, so we included risk factors for DVT throughout the perioperative period, which increased the reliability of the model to some extent. In summary, the specialized DVT risk assessment models were regarded as research hotspots, but there were few models for venous thrombosis in patients with bone trauma.

In this study, perioperative RBC infusion was a predictor of postoperative DVT in patients with femoral fracture. Rothstein, Goel et al. [35–37] also confirmed that perioperative RBC infusion increases the risk of VTE. A retrospective cohort study of 1233 trauma patients by Meizoso et al. [38] found that transfusion of more than 4 units was an independent risk factor for DVT. The transfusion may increase blood viscosity and change local hemorheology, leading to aggregation of RBC [39]. In addition, the increased number of RBC may lead to the aggregation of platelets [40], RBC infusion may induce an inflammatory response, which may further lead to postoperative thrombosis in patients [41]. For bone trauma patients with high risk factors for DVT, we might be more strict about the indications for blood transfusion. At the same time, the monitoring and prevention of DVT should be strengthened in such patients receiving RBC infusion. The nephropathy was included in the predictive model, and other studies have confirmed a correlation between chronic kidney disease and DVT [27, 42, 43]. The reason for the higher risk of DVT in patients with renal disease is unknown. The inflammatory state associated with chronic renal disease may induce hypercoagulability [42].Complications of renal disease or therapeutic drugs (hormonal drugs, immunosuppressants) may also adversely affect the development of DVT [43]. Zuo et al. [44] found that hypoalbuminemia was an independent risk factor for DVT in patients with bone trauma, and hypoalbuminemia was one of the predictors in our model. We consider that hypoalbuminemia may be a reflection of trauma severity which is associated with increased albumin catabolism [45]. The decrease of albumin causes the disorder of plasma osmotic pressure and serum composition, and may also be related to the loss of liver function, thus increasing the risk of death in DVT patients [46]. The preoperative improvement of hypoalbuminemia in patients with bone trauma may further reduce the risk of DVT. In this study, COPD was an independent risk factor for DVT and was included in the model. COPD patients are usually accompanied by chronic hypoxia, poor lung function, and decreased activity tolerance, resulting in slow blood flow and high blood coagulation [32, 47, 48]. Therefore, we should pay attention to preoperative optimization of lung disease in patients with bone trauma. Therefore, we should pay attention to the optimization of comorbidities in patients with bone trauma, and conduct early preoperative intervention according to preoperative conditions and laboratory test indicators. For example, correcting hypoalbuminemia, improving lung function in COPD patients, and avoiding aggravating renal damage in patients with renal disease may reduce the risk of DVT. The trauma to surgery > 7 days was another predictor of this study. Song et al. [49] reported that > 7 days after femoral neck fracture increased the risk of preoperative DVT by 2 times. In patients with pelvic and acetabular fractures, trauma to surgery > 2 weeks was an independent risk factor for DVT [50]. A multicenter study also showed a close association with early thrombosis within 72 h after trauma [51]. Therefore, shortening the time from trauma to surgery is an important factor in minimizing the risk of DVT development.. We can shorten the time to transfer to a higher level hospital for treatment due to critical illness or medical conditions. Physicians should make individualized preoperative preparation plans for patients with medical complications. In addition, we need to reduce unnecessary preoperative examinations. To a certain extent, these measures allow patients to undergo surgery as early as possible. The CRP level on day 3 after surgery was one of the predictors in nomogram model. This might be due to the interaction between inflammation and coagulation. On the one hand, inflammation activates coagulation, and on the other hand, coagulation significantly affects the inflammatory response [52]. Complement activation enhances coagulation by increasing tissue factor expression and inhibiting fibrinolysis [53].The inflammatory cytokines can bring down the anticoagulant activity of the protein C pathway [54].The CRP is an acute phase protein that is rapidly synthesized in the liver, mainly in response to the stimulation of inflammatory cytokines and CRP levels drop rapidly when inflammatory cytokine stimulation is attenuated [55]. Acute inflammation reflected by CRP is a trigger for VTE [56]. However, NEUT% and WBC do not have the above characteristics of CRP, which may be the reason why they are not independent risk factors for DVT. A prospective study of CRP levels within 7 days after surgery in patients with cardiac, vascular, abdominal, and thoracic surgery reported a peak CRP level on day 3 after surgery in uninfected patients [57].Combined with our results, it might be that the third day after surgery was the peak period of inflammatory response in patients with bone trauma. There is no consensus on many issues with anticoagulation. Few studies have investigated the timing of postoperative anticoagulation in patients with bone trauma. This study found that early postoperative anticoagulation might reduce the risk of DVT, but we should weigh the benefits of early postoperative anticoagulation against the increased risk of bleeding. Combined with the related research on the risk factors of DVT in patients with bone trauma in recent years, the reliability of the predictive factors in the nomogram model has finally been verified.

At present, the nomogram is widely used in medical treatment, especially the establishment of clinical prediction models [58, 59].The nomogram transforms complex regression equations into visual graphs, which makes the results more intuitive. At present, there are not many models for predicting the risk of perioperative DVT in patients with bone trauma. We established a nomogram prediction model of postoperative DVT risk for patients with femoral fracture, which provided a certain reference for the specialized assessment of DVT risk in patients with bone trauma. However, there are some limitations in our study. Any retrospective analysis may be subject to errors due to selection bias. However, we reduced selection bias by including subjects in consecutive time periods. In addition, we only carried out internal verification, and we can carry out multicenter research and increase the sample size to complete external verification in the later stage, so as to further improve the model.

## Conclusions

Based on the risk factors for DVT throughout the perioperative period, we established a nomogram model that effectively predicts postoperative DVT in patients with femoral fracture. This model is a reference for identifying patients at high risk of DVT, and early optimization of relevant risk factors can further reduce the incidence of DVT. In future, it is indeed important to establish more specialized models that can provide references for formulating individualized prevention strategies for DVT in patients with specific conditions, such as bone trauma. These specialized models should take into account the unique risk factors and characteristics associated with the particular patient population.

## Data Availability

All the data will be available upon motivated request to the corresponding author of the present paper.

## Abbreviations

DVT: Deep vein thrombosis
VTE: Venous thromboembolism
ACCP: American College of Chest Physicians
BMI: Body mass index
COPD: Chronic obstructive pulmonary disease
ASA: American Society of Anesthesiologists
RBC: Red blood cell
CRP: C-creative protein
Crea: Creatinine
Alb: Albumin
WBC: White blood cells
Hb: Hemoglobin
PLT: Platelet counts
NEUT%: Neutrophil percentage
LYM%: Lymphocyte percentage
PT: Prothrombin time
PTR: Prothrombin time ratio
INR: International normalized ratio
PTA: Prothrombin time activity
APTT: Activated partial thromboplastin time
TT: Thrombin time
Fbg: Fibrinogen
DD: D-dimer
FDP: Fibrinogen degradation products
Lac: Lactic acid
ROC: Receiver operating characteristic
AUC: Area under the curve
DCA: Decision curve analysis
